# The Use of PET-CT in the Assessment of Patients with Colorectal Carcinoma

**DOI:** 10.1155/2011/846512

**Published:** 2011-07-03

**Authors:** Owen J. O'Connor, Shanaugh McDermott, James Slattery, Dushyant Sahani, Michael A. Blake

**Affiliations:** Abdominal Imaging & Intervention Division, Massachusetts General Hospital, 55 Fruit Street, Boston, MA 02114, USA

## Abstract

Colorectal cancer is the third most commonly diagnosed cancer, accounting for 53,219 deaths in 2007 and an estimated 146,970 new cases in the USA during 2009. The combination of FDG PET and CT has proven to be of great benefit for the assessment of colorectal cancer. This is most evident in the detection of occult metastases, particularly intra- or extrahepatic sites of disease, that would preclude a curative procedure or in the detection of local recurrence. FDG PET is generally not used for the diagnosis of colorectal cancer although there are circumstances where PET-CT may make the initial diagnosis, particularly with its more widespread use. In addition, precancerous adenomatous polyps can also be detected incidentally on whole-body images performed for other indications; sensitivity increases with increasing polyp size. False-negative FDG PET findings have been reported with mucinous adenocarcinoma, and false-positive findings have been reported due to inflammatory conditions such as diverticulitis, colitis, and postoperative scarring. Therefore, detailed evaluation of the CT component of a PET/CT exam, including assessment of the entire colon, is essential.

## 1. Manuscript

Colorectal cancer (CRC) is a major cause of cancer-related mortality in Western countries. It is the third most commonly diagnosed cancer, with an estimated 146, 970 new cases diagnosed in the USA during 2009 [1]. In 2007, the annual mortality rate from colorectal cancer was 20.0 and 14.1 per 100,000 in men and women, respectively, accounting for 53,219 deaths [2]. Approximately 80% of patients present with locoregional disease and 20% with metastatic disease [3]. Positron emission tomography-computed tomography (PET-CT) is of considerable assistance to clinicians in the management of patients with CRC.

## 2. PET-CT for the Initial Diagnosis of CRC

**Figure 1:**
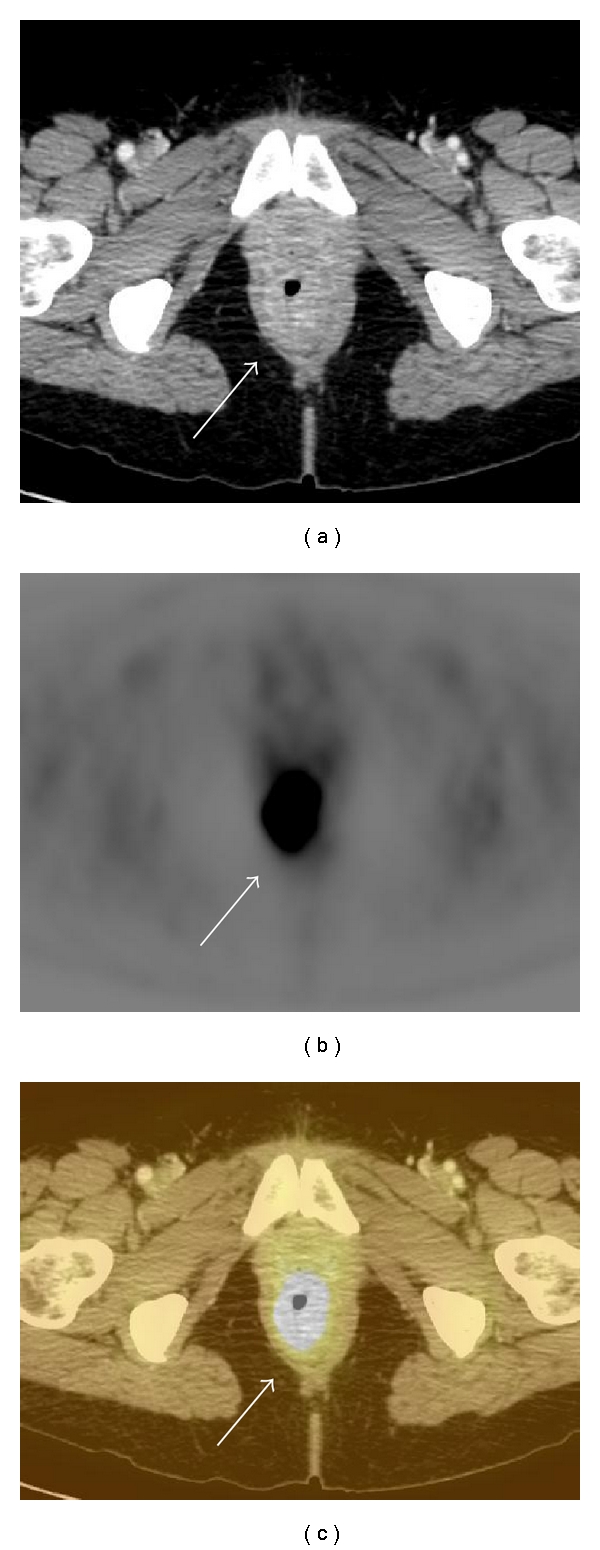
Rectal carcinoma depicted on PET-CT. (a) There is circumferential rectal thickening (arrow) on contrast-enhanced CT. (b) There is avid FDG radiotracer uptake (arrow) on axial FDG-PET. (c) Increased FDG uptake on FDG-PET correlates with thickening (arrow) due to rectal carcinoma seen on CT.

In practice, FDG PET is rarely used for the primary diagnosis of CRC although there are circumstances where PET-CT makes the initial diagnosis (Figure 1). 

**Figure 2:**
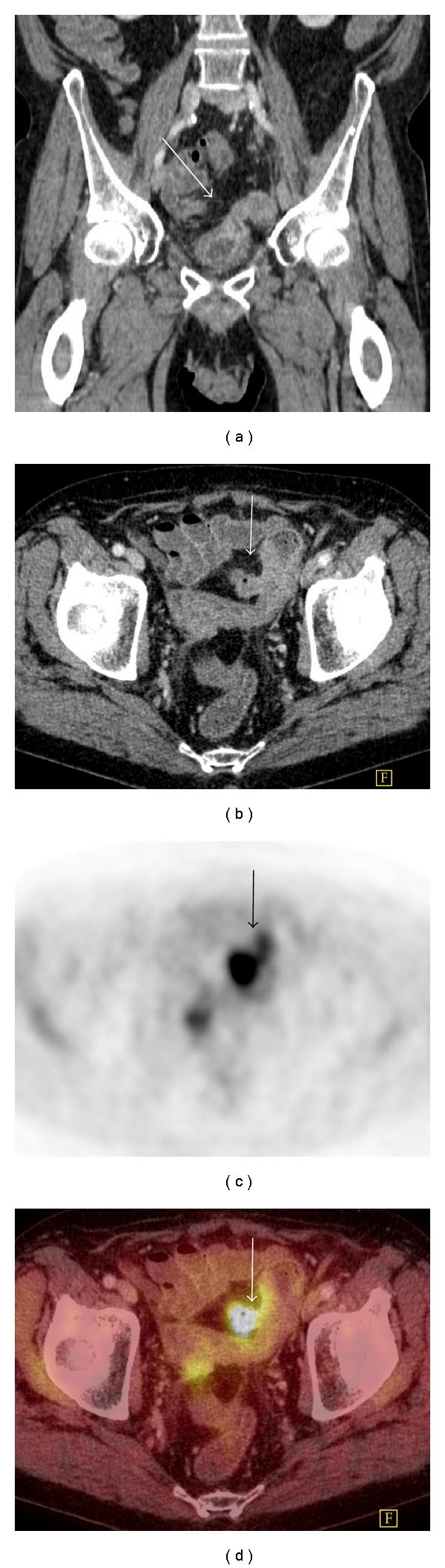
Colovesical fistula in a patient with a prior history of anterior resection for rectal carcinoma. (a) There is a connection (arrow) between the colon and bladder on coronal CT. (b) The colonic end of the fistulous tract (arrow) is seen on axial CT. (c) There is a focus of avid FDG-PET uptake (arrow) present. (d) Fused images confirm that the uptake is within the fistula (arrow) and probably represents urine containing FDG within the fistulous tract. The presence of the fistula and the absence of tumor were confirmed at cystoscopy.

Precancerous adenomatous polyps can also be detected incidentally on whole-body images performed for other indications. The sensitivity of PET-CT in this setting improves with increasing polyp size [4, 5]. CT can help distinguish benign from malignant causes of abnormal FDG uptake, and combined PET-CT clearly performs better than either modality in isolation (Figure 2).

**Figure 3:**
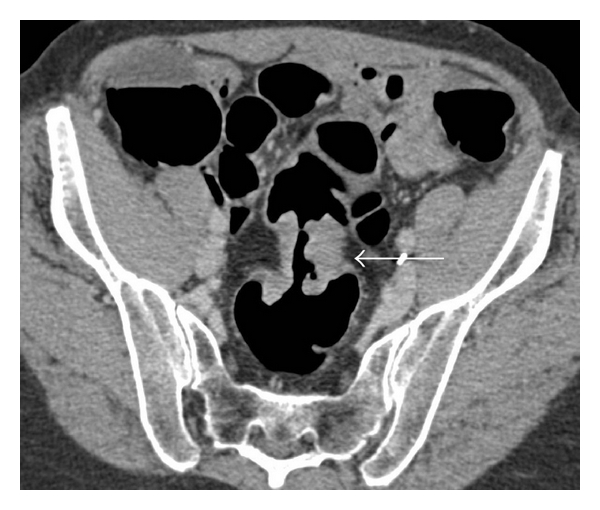
Carcinoma at the rectosigmoid junction at CT colonography. There is enhancing tissue at the rectosigmoid junction with circumferential wall thickening (arrow), shouldered margins, and luminal stenosis consistent with carcinoma.

Conventional CT is limited in the detection of subtle colonic polyps and tumors although contrast-enhanced CT with oral contrast can be helpful in detecting gross masses and assessing their complications. CT colonography with a distended, clean colon is now accepted as a screening test for CRC with a high accuracy for detecting both adenomatous polyps and malignant tumors (Figure 3) [6]. 

**Figure 4:**
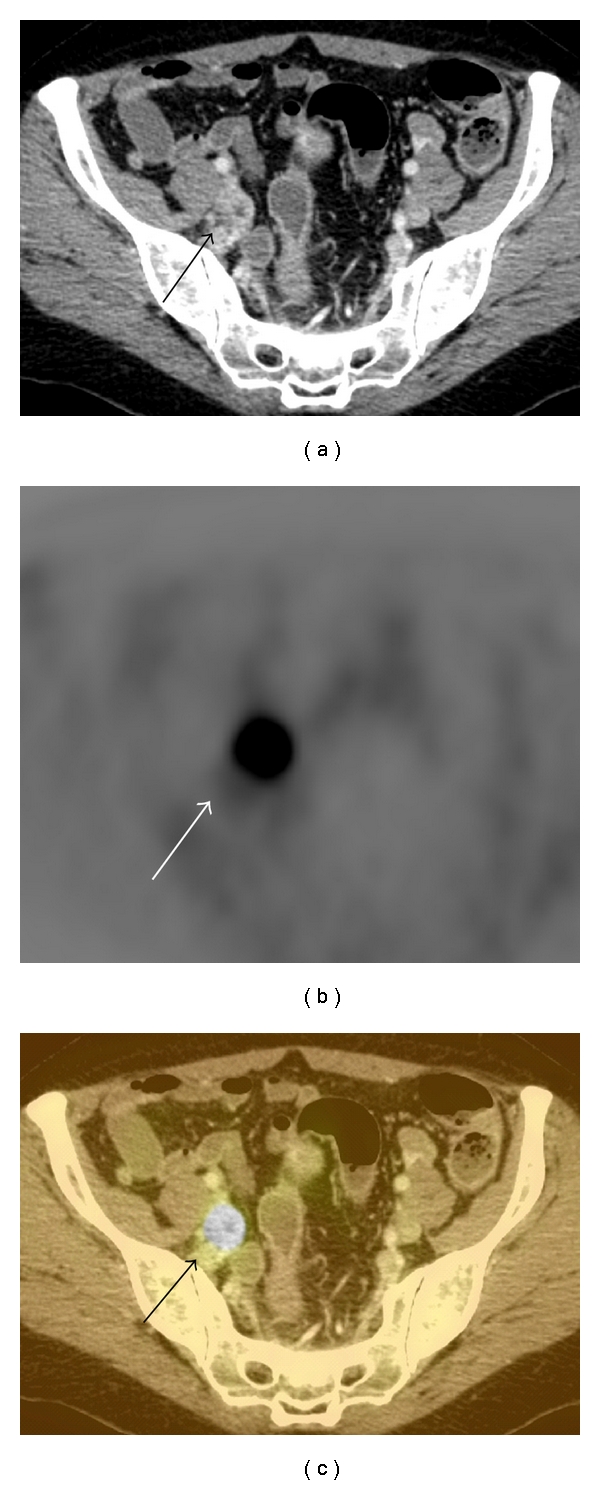
Rectal carcinoma with spread to the internal iliac chain lymph nodes. (a) There is an abnormally enhancing iliac lymph node (arrow) on contrast-enhanced CT. (b) There is avid radiotracer uptake (arrow) on the right side of the pelvis on FDG-PET (arrow). (c) Increased uptake on FDG-PET (arrow) correlates with the lymph node lesion seen on CT and is consistent with a malignant deposit.

The sensitivity of CT colonography for the detection of CRC is approximately 95% which compares very favorably with that of barium enema (94%) and optical colonoscopy (92%) [7]. PET-CT colonography, while being an intriguing prospect, may have too great a radiation load for a screening study but may yet prove appropriate for selected indications [8]. CT colonography and indeed all CT applications have benefited from the recent dramatic advances in CT technology with multidetector scanners now up to 320 slice allowing isotropic imaging, increased speed, resolution, and 3D reconstructions which referring physicians can also find helpful. CT radiation dose optimization is also now a more realizable goal with the development of various dose reduction strategies such as automated iterative reconstruction techniques and automatic tube current modulation (Figure 4) [9]. 

## 3. PET-CT for the Staging of CRC (T Staging)

Accurate preoperative staging is essential to estimate prognosis and institute appropriate therapy in CRC. 

 Locoregional staging of CRC by imaging can be performed using transrectal ultrasound, magnetic resonance imaging (MRI), and CT while metastatic disease is best assessed with CT, MRI, 18-flourine fluorodeoxyglucose (FDG) PET, and more recently PET-CT. FDG PET is unsuitable for precise local and regional staging primarily due to its limited spatial resolution, where precise depth of invasion is the primary determinant. Therefore, current staging schemes are based on surgical and pathologic data. PET may be accurate in selected cases of gross serosal penetration and local invasion. CT allows more precise structural information than PET, but usually cannot discriminate bowel wall layers in the manner of MRI or endoscopic US. A meta-analysis from 2004 of endoscopic US, CT, and MRI for the assessment of perirectal invasion by CRC reported a significantly higher sensitivity estimate for US than for CT (*P* < .001) and MR imaging (*P* = .003), indicating understaging of T3 (or higher) tumors with CT and MR imaging (Table 1). The specificity estimates for US, CT, and MR imaging as well as the sensitivity and specificity of US, CT, and MRI for the detection of adjacent organ invasion were reported as comparable [10]. 

**Figure 5:**
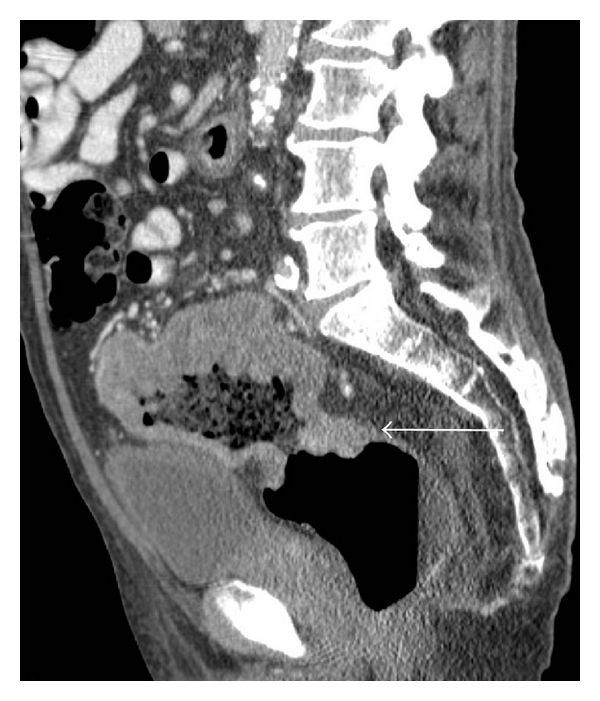
Large upper rectal adenocarcinoma with possible involvement of the bladder wall there is a large aneurysm-type carcinoma (arrow) of the rectum present with stranding of the fat between the tumor and the bladder which is concerning for bladder involvement which would classify the tumor as T4.

T staging of CRC by PET-CT is thus almost exclusively CT dependent. T staging of rectal malignancy has seen many advances in recent years. In the setting of rectal cancer, endorectal ultrasound is very accurate for staging superficial T1 and T2 tumors but less so for T3 and T4 tumors [11]. The imaging of T3 and T4 tumors is better performed with either MRI or CT (Figure 5). Recent studies have shown MRI using a phased array coil to be highly accurate and the imaging method of choice for staging primary rectal tumors and assessing for involvement of the mesorectal fascia [12]. Preoperative MRI is not feasible in all patients, and multislice CT is a potential “one-stop” method of imaging the mesorectum and identifying distant metastases. The accuracy of multislice CT for the evaluation of mesorectal extension by rectal carcinoma is dependent on the site of the primary tumor. Overall sensitivity and specificity for predicting mesorectal fascial involvement using CT are 74% and 94%, respectively. For tumors in the lower third of the rectum the sensitivity and specificity are reduced at 66% and 82%, respectively. Sensitivity and specificity of CT for assessing mesorectal extension are much better for tumors of the upper two-thirds of the rectum (76% and 96%, resp.), mainly due to the more generous amount of perirectal fat enveloped by the mesorectal fascia at higher levels [13].

## 4. PET-CT for the Staging of CRC (N Staging)

Over 75% of patients with CRC have disease limited to the bowel or regional pericolic or mesenteric lymph nodes at diagnosis [14]. Therefore, most surgeries are performed with curative intent. Regional lymph node involvement is best determined by surgical-pathologic resection and analysis. Preoperative nodal staging using imaging requires an assessment of the number of pericolic and mesenteric nodes containing metastatic disease. False-negative PET findings in regional metastatic lymph nodes are in part due to the intense FDG uptake by the primary site, which obscures the immediately adjacent structures, and also due to PET's low sensitivity for microscopically involved lymph nodes. For these reasons, FDG PET has been found to be only 29% sensitive for regional lymph node metastases from CRC, whereas the specificity of FDG PET is 96% [15, 16]. Enlarged and nonenlarged FDG avid lymph nodes can be identified in the mesentery on PET-CT, indicating the presence of regional lymph node metastases, but this is more commonly encountered on restaging patients with CRC rather than on initial staging. The presence of only regional lymph node metastases on PET-CT performed for initial staging would not in any event preclude resection of the primary lesion. Similarly, local regional nodes are resected by surgical excision nonetheless, an attempt to detect nodal or organ metastases preoperatively using PET-CT is important in planning the general therapeutic approach (i.e., palliation versus curative tumor resection). Furthermore, it has been reported that PET-CT can alter the staging of CRC compared with conventional imaging in 31% of cases [17]. Altered staging as a result of PET-CT changes treatment intent in 8% of cases, mainly due to revised nodal staging (Table 4).

## 5. PET-CT for the Staging of CRC (Metastatic Disease)

### 5.1. Hepatic Metastases

**Figure 6:**
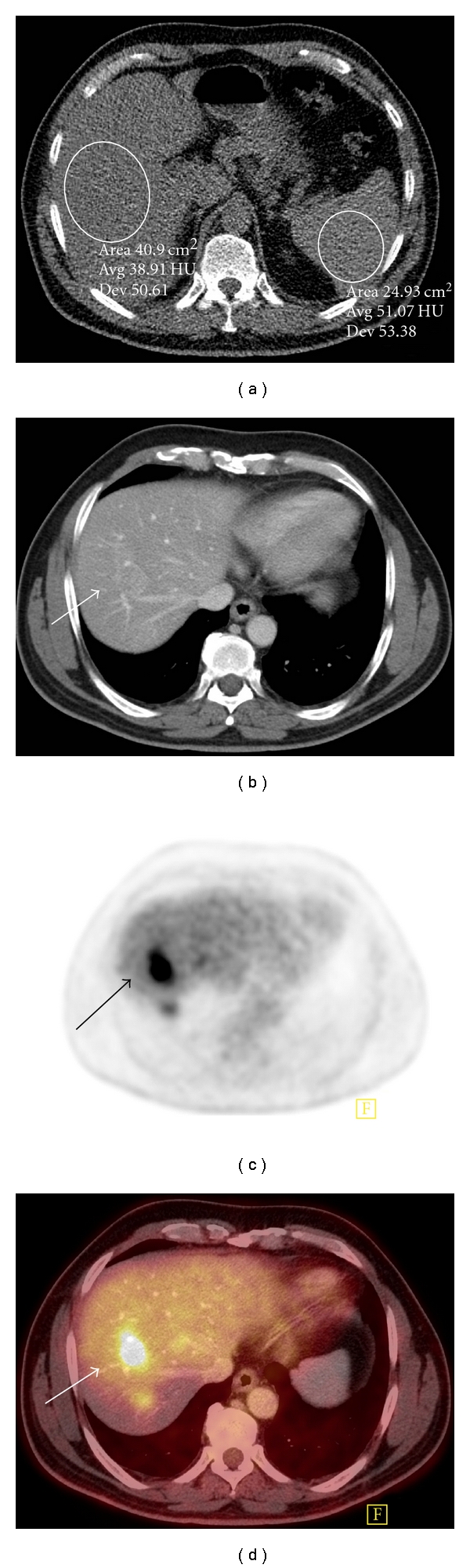
Liver metastatic deposit in a fatty liver that is difficult to appreciate on CT. (a) There is diffuse fatty infiltration of the liver on unenhanced CT with attenuation values in the liver far lower than those of the spleen. (b) There is a very subtle lesion (arrow) in segment 7 on contrast-enhanced CT imaging. (c) There is avid radiotracer uptake (arrow) in the right lobe of liver FDG-PET. (d) Increased uptake on FDG-PET (arrow) correlates with the subtle liver lesion on fused PET-CT consistent with a hepatic metastasis.

Carcinoma that has spread outside regional pericolic or mesenteric lymph nodes is considered metastatic. Sites of metastatic spread include liver, retroperitoneal, and iliac lymph nodes, bone, adrenal glands, and soft tissue. Contrast-enhanced multidetector-row CT is the primary imaging modality for the detection, localization, and characterization of focal liver lesions in many hospitals [18]. The present authors advocate PET-CT be acquired using an intravenous (IV) contrast-enhanced portal-venous phase, fully diagnostic quality CT. This method maximizes contrast resolution for detection of relatively low attenuation hepatic metastases, which is lacking in the low-dose non-IV contrast CT (Figure 6).

This is of particular importance in mucinous metastases which can be poorly FDG avid. Moreover, portal-venous phase contrast-enhanced CT optimizes vessel and hepatic segment delineation, should the patient be a candidate for liver resection surgery. 

Given the known benefits of such appropriate liver metastasectomy for patients with CRC and metastases confined to the liver, PET-CT plays an important role by detecting additional extrahepatic sites of disease that would preclude a curative procedure. Several investigators have examined the incremental value of FDG PET as a supplement to CT and found that FDG PET offers information beyond that from CT alone and that this information often affects patient care. FDG PET combined with CT for preoperative planning in patients with hepatic metastases identifies additional sites of extrahepatic disease in 11–32% of patients [19–21]. This frequently changes management from a localized treatment strategy to a more systemic approach with chemotherapy. 

FDG PET is highly sensitive for the detection of liver metastases [22–25]. A recent meta-analysis demonstrated the sensitivities of CT, MR, and FDG PET for detecting hepatic metastases from CRC to be 83.6%, 88.2%, and 94.1%, respectively, [25] (Table 2). The sensitivity of MR is improving with the development of newer MR technologies, and modern MR is superior to CT for detecting lesions which measure less than 1 cm [25]. PET is accurate for identification of hepatic metastases greater than 1 cm in diameter but is limited in its ability to demonstrate lesions smaller than 1 cm [26–28]. PET-CT imaging may be particularly useful in patients with several hypodense or hypoenhancing liver lesions that are not clearly characterized by CT alone and in patients in whom standard CT fails to detect metastases in the setting of a rising serum CEA. In these cases, PET-CT can direct patient management by guiding biopsies or ablations or directing surgical resections of liver metastases.

FDG-PET and PET-CT can help clarify abnormal CT findings and depict otherwise unsuspected metastases to nonenlarged lymph nodes. PET-CT can often find sites of disease in patients with abnormal carcinoembryonic antigen (CEA) levels and prior negative workup with conventional diagnostic modalities. A recent study of PET-CT for the detection of recurrent or metastatic disease in patients with a history of CRC and raised CEA found PET-CT to be considerably better than multislice CT alone [29] (Table 3). PET-CT for preoperative staging has been shown to improve survival in patients with hepatic metastases deemed suitable for hepatic resection mainly again by demonstrating the presence of extrahepatic metastases, thus appropriately eliminating ineffective surgery in patients with inoperable disease [19]. Indeed, FDG PET in patients with CRC is a cost-effective technique that often changes patient management [30]. Early identification of liver metastases provides the opportunity for neoadjuvant chemotherapy and resection, which again may prolong survival in such patients with CRC [23, 31].

Liver metastases from CRC represent 50% of all recurrences. The size and number of hepatic metastases and the presence of extrahepatic disease affect the prognosis. Untreated patients with limited metastases to the liver have a 5-year survival rate of 28% and long-term survival with chemotherapy alone is equally dismal [32, 33]. Surgical resection is the only potentially curative therapy in these patients. Patients most suited to undergo liver resection are those with fewer than four lesions, smaller than 5 cm, and without extrahepatic disease [34]. Recurrence rates after resection of liver metastases are high however, and there is significant associated morbidity and mortality. Surgical treatment offers 5-year survival rates of 25% to 37% and 10-year survival rates of 20% [35–37].

### 5.2. Lymph Node Metastases

CRC spread to lymph nodes beyond regional pericolic or mesenteric lymph nodes is considered metastatic spread (M1 disease). CT's strengths are its accurate depiction of anatomic abnormalities and its ability to define structures that are below the resolution of conventional nuclear medicine imaging. A major limitation of CT, however, is its reliance on anatomic criteria in order to identify pathologic conditions, for example using a 1 cm short-axis dimension threshold for pathologic lymphadenopathy. Although this criterion usually results in an acceptable balance between sensitivity and specificity for disease, nodes smaller than 1 cm can contain malignant cells and, in addition, reactive or inflammatory processes can result in nodes larger than 1 cm. Furthermore the upper limit of what should be considered normal sized lymph nodes by CT varies with the specific location but, in general, is accepted to be 10 mm in short axis for retroperitoneal, mesenteric, external iliac, and inguinal nodes, 8 mm for internal iliac, obturator, and lateral sacral nodes and 5 mm for perirectal nodes. Other overlapping CT nodal features that can be seen with lymph node metastases and inflammatory conditions include low density (as in mucinous metastases) and calcifications. 

Nodal metabolic activity assessment using FDG PET is not directly reliant on nodal size to determine the presence or absence of malignancy. Nodes that are not enlarged can contain tumor on FDG PET images, and nodes that are enlarged can be shown to be reactive in nature. For this reason, PET is more sensitive and specific than CT alone for identification of lymph node metastases [38]. However, PET also has limitations regarding size, as false-negatives can occur in small lymph nodes and false-positives may occur due to inflammation. In addition some cystic or mucinous nodal metastases may not show significant FDG uptake, and hence the FDG PET images cannot be entirely relied upon to exclude lymph node metastases in the setting of CRC.

### 5.3. Pulmonary Metastases

**Figure 7:**
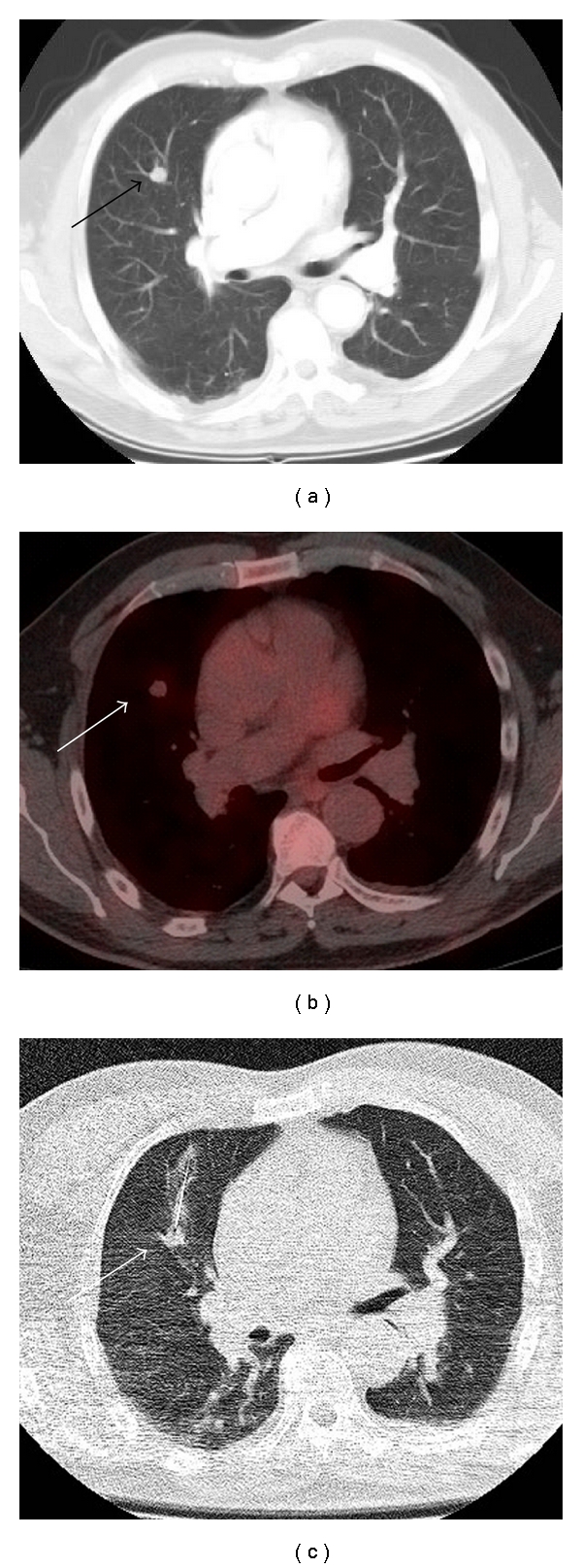
Patient with rectal carcinoma and mildly FDG avid metastases to lung. (a) There is a suspicious right upper lobe lung nodule (arrow) on CT in a patient with prior history of rectal carcinoma. (b) The lesion is only mildly FDG avid (arrow) on fused PET-CT. Based on these images the lesion is not definitively malignant. This may be due to the limited resolution of PET and the small lesion size or reduced cellularity. (c) Lung biopsy was subsequently performed confirming that the lesion (arrow) represented a metastatic deposit from rectal carcinoma.

The lung is the second most common organ for colorectal metastatic diseases. Pulmonary metastatic disease manifests as pulmonary nodules, lymphangitic carcinomatosis, and thickening of the pulmonary interstitium or of the pleura. Ten percent of patients with CRC develop lung metastases, usually in association with other extrathoracic systemic disease. But 2–4% will have isolated lung metastases and 50% of these will be amenable to resection [39, 40]. The sensitivity and specificity of PET-CT for the detection of malignant solitary pulmonary nodules are 96% and 83%, respectively, [41]. Peroperative morbidity is low, and 5-year survival ranges from 28% to 40% after successful resection [39, 40, 42, 43]. The CT component of PET-CT provides high resolution and is thus the first choice for lung nodule detection, and the FDG PET component improves specificity for nodules generally greater than 8 mm. Standard chest CT is now able to delineate pulmonary nodules as small as 2-3 mm. This facilitates the detection of even the smallest pulmonary nodules, leading to a therapeutic dilemma because the low specificity of such findings (most pulmonary nodules will be benign) remains a problem [44] (Figure 7). Furthermore, in daily practice, atypical imaging features of metastases are often encountered making a distinction of metastases from other benign pulmonary diseases based on CT findings alone difficult. Due to limited spatial resolution, a negative PET scan cannot exclude pulmonary metastases with certainty, while a positive FDG-PET will generally confirm suspicious abnormalities detected on the CT images [45]. A standardized uptake value (SUV) of greater than 2.5 suggests that a solitary pulmonary nodule is malignant with a relatively high degree of sensitivity and specificity [46]. Known nonmalignant causes of increased FDG uptake in the lung parenchyma include inflammatory processes such as granulomatous disease, cryptogenic organizing pneumonia, tuberculosis, and aspergilloma, while previous talc pleurodesis can result in false-positive uptake in the pleura [47]. Respiratory gating during the acquisition of PET-CT shows potential for better spatial resolution on FDG-PET and improved correlation between nodule SUV values and nodule size [48].

### 5.4. Osseous Metastases

Osseous metastases from CRC are relatively uncommon. A review of over 5,000 cases reported osseous metastases combined with visceral metastases in 6.6% and isolated skeletal metastases in 1.1% of cases [49]. Osseous metastases may be overlooked on CT particularly if metastatic disease is confined to the skeleton or if the patient has coexistent osteoporosis. Typical appearances of bone metastases on CT include lytic or mixed lytic/sclerotic lesions which can become sclerotic with treatment. FDG uptake in bone marrow is usually roughly equivalent to, or slightly greater than, that of liver background tracer activity. FDG uptake in marrow of the vertebral bodies can appear focal on axial imaging and can be misinterpreted. A repeating regular pattern usually best appreciated on sagittal or coronal images is characteristic of physiologic marrow FDG uptake. Metastases are distinguished by more intense FDG uptake and a nonuniform distribution. Patients undergoing treatment with hematopoietic stimulates as part of their chemotherapy regimen can demonstrate uniformly high marrow FDG accumulation, and thus PET imaging ideally should not be scheduled immediately after their administration [50]. 

Studies suggest that PET-CT is both sensitive and specific for diagnosing malignant bone lesions [51]. For example, one study of 712 patients, 59 of whom had bone metastases, demonstrated PET/CT to have a positive predictive value of 98% when findings on PET and CT were concordant [52]. Focal FDG uptake on PET often draws attention to subtle bony changes of early metastases on the CT. Occasionally no corresponding CT abnormality is seen and if deemed necessary MRI may be helpful to clarify discordant PET-CT findings. Increased osseous FDG uptake can occur secondary to fractures, hemangiomata, or increased hematopoietic activity in patients receiving chemotherapy [53].

### 5.5. Other Soft Tissue Metastases 

Peritoneal implants due to colon cancer can be difficult to detect on CT alone particularly in cachectic patients without careful attention to technique and interpretation. FDG PET images are very useful for locating peritoneal implants 7-8 mm or greater in size. The adrenal glands are another favored site for colorectal metastases for which PET imaging is very sensitive. Adrenal adenomas can uncommonly exhibit increased FDG activity, and all of the reported cases of PET-positive adenomas have been indeterminate by CT criteria (>10 HU). Analysis using CT contrast washout analysis may be useful for further lesion characterization but adrenal biopsy is the final arbiter for metastases if histologic confirmation is required.

## 6. Restaging of CRC

Locoregional pelvic recurrence and liver metastases are the major sites of relapse after resection of CRC. Recurrence occurs in one third of patients in the first 2 years after resection. Patterns of recurrence are different in colon cancer compared with rectal cancer. Local recurrence is more common in patients with rectal rather than colon cancer ranging from 7% to 33% and 1–19%, respectively, [54]. Only 20% of local recurrences can be completely resected, and most patients who develop local recurrence die from their disease [55]. Several studies have reported on the spread and survival patterns regarding local, locoregional, and metastatic recurrence. Willett et al. reviewed recurrent colon carcinoma, finding that 20% of recurrences were local failures only and 43% were concurrent local and distant [56]. Pilipshen et al. found that 30% of the recurrences were pelvic alone and 58% were both pelvic and distant [57]. Tumor stage, perforation, local invasion, and rectal carcinoma are positive predictors of recurrence [58]. Brethauer et al. reported that 54% of recurrences had distant metastases alone at the time of recurrence and 67% had distant metastases as a component of failure [55]. 

Focal FDG uptake guides radiologists to examine the corresponding CT for concordant abnormalities such as a soft tissue mass or bone erosion on CT. Correlation with a patient's clinical status is also vital in PET-CT interpretation as some imaging features overlap between abscess and cystic metastatic disease. Mucinous adenocarcinoma of the colon can cause widespread intraperitoneal metastases, which can be detected with CT only if they produce thickening of the peritoneal surfaces or peritoneal nodules. Microscopic seeding of the peritoneal surfaces will not be detected by either CT or PET. However, the combination of CT and PET together can help identify macroscopic peritoneal implants.

**Figure 8:**
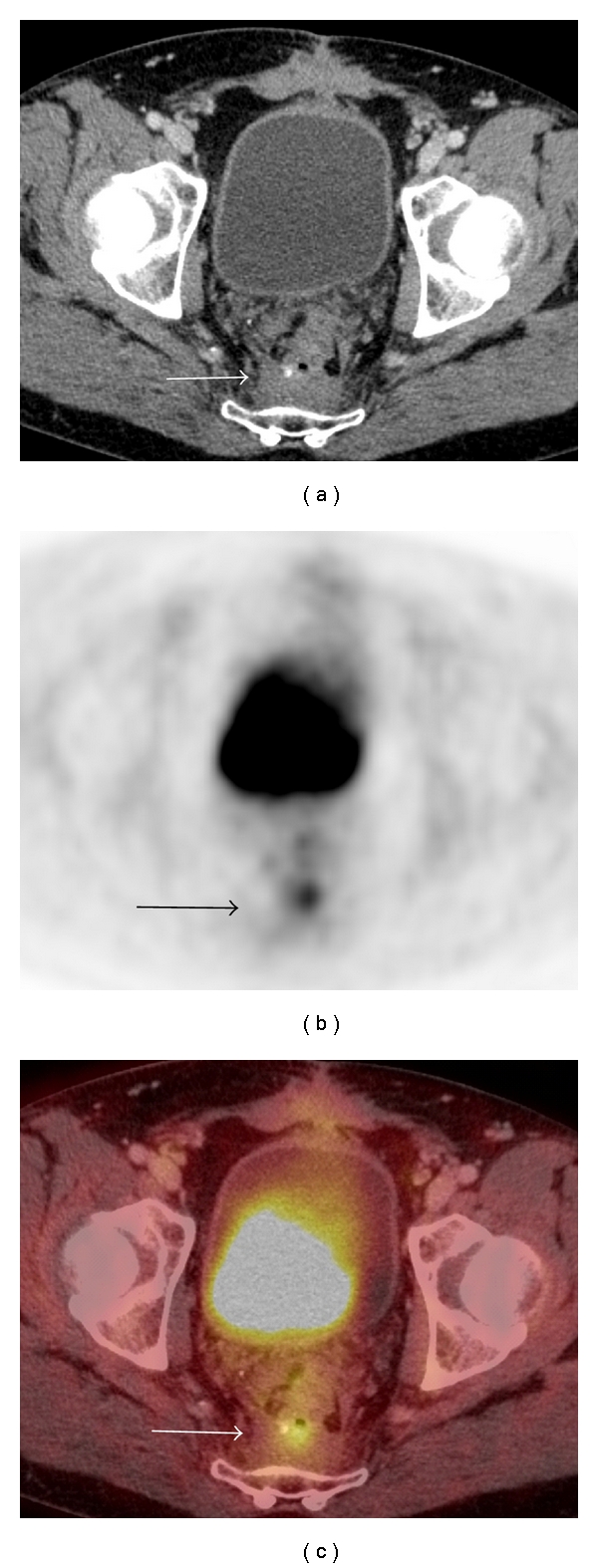
Local recurrence 2 years following resection of rectal carcinoma. (a) There is soft tissue material in the presacral space (arrow) that may be postoperative in nature but the presence of malignancy is difficult to exclude on CT alone. (b) There is a small focus of avid radiotracer uptake (arrow) on the on FDG-PET. (c) Increased uptake on FDG-PET (arrow) correlates with the presacral soft tissue (arrow) and is characteristic of tumor recurrence in a patient who has not had recent surgery in this area and has no symptoms of leak or infection.

Differentiating the posttherapy appearances such as surgical scarring and radiation fibrosis from disease recurrence is particularly challenging in patients with CRC. This problem is mostly encountered with distal colon and rectal tumors, where presacral scarring and pelvic changes are common. Serial examinations are frequently required using conventional imaging, before slowly developing changes can be appreciated over time. Metabolic activity in the presacral space is indicative of tumor recurrence on FDG PET performed 6 months following surgery, while postsurgical change is not hypermetabolic (Figure 8). FDG PET is accurate for differentiation of benign from malignant presacral changes and is superior to CT and MR imaging in this regard [59]. Metabolic imaging has a further advantage in that only a single study is necessary to make this determination, rather than the serial morphologic studies often required with conventional imaging. However, PET alone has limitations given the anatomical changes in appearance and position seen resulting from surgery. Even-Sapir et al. documented the additional value of PET-CT in the detection of pelvic recurrence 6 months or longer after surgical removal of rectal cancer [60]. PET-CT imaging has the potential to become the preferred imaging modality in these patients, because it identifies and localizes the disease in one setting and can guide diagnostic or therapeutic interventions [61]. Surgical management of local recurrence depends on the site and extent of the disease. PET-CT can be particularly useful for demonstrating recurrent rectal cancer involving adjacent pelvic organs, pelvic sidewalls, or sacrum and for planning potential pelvic exenteration, en bloc resection, or palliative diversion procedures.

Wound recurrences are rare and, although resectable when isolated, are usually indicative of diffuse intraabdominal disease. Isolated recurrence can occur at the site of prior resection of metastases and PET-CT can be helpful in precisely locating and characterizing such recurrences. Similarly, anastomotic recurrences may be resected but can be associated with more extensive local or metastatic disease, demonstrable by PET/CT, which would usually preclude resection.

## 7. Reporting of PET-CT in CRC

Dictating a concise and pertinent PET-CT report of a study performed on a patient with CRC firstly requires an accurate clinical history be available so that individual specific questions can be addressed. Relevant issues include the patient's preoperative or postoperative status, prior imaging, and colonoscopic findings. The oncologist often wants to know of findings pertinent to staging or restaging and the development of complications from the tumor or issues requiring intervention such as abscess, venous thrombosis, bowel obstruction, perforation or urinary tract obstruction. The surgeon may seek specific factors that will influence potential operations including vascular relationships or postoperative complications. Relevant nonneoplastic findings should also be recorded. Pertinent negatives and positives should be detailed in the body of the report, and specific issues, conclusions, and recommendations stated in the impression. Important or unexpected findings should be directly communicated to the referring physician.

In patients without a known history of CRC, it is important to be aware that colonic FDG uptake may be quite avid in the absence of pathology particularly in the cecum/ascending colon and within the rectoanal region. Moreover, inflammatory conditions such as Crohn's disease, ulcerative colitis, typhlitis, and diverticulitis can all lead to increased colonic FDG activity. Regardless of etiology, however, focal large bowel activity greater than hepatic activity is unusual and should alert the interpreting physician to the possible presence of pathology. A thorough regional review of coregistered CT images is warranted to assess for focal masses or neighboring signs of inflammation bearing in mind that peristalsis, patient motion, and breathing may lead to misregistration of the PET and CT images. A long segment of diffusely increased colonic FDG uptake should not be interpreted as inflammatory bowel disease unless there are coexistent CT findings as this pattern of uptake is not uncommonly seen. In the absence of specific corresponding benign CT findings, focal intense colonic activity on PET usually warrants further investigation such as colonoscopy.

## 8. False-Negative FDG-PET Findings

As mentioned earlier, false-negative FDG PET findings have been reported with mucinous adenocarcinoma since the sensitivity of FDG-PET for mucinous adenocarcinoma is significantly lower than that for nonmucinous disease [62]. Detailed diagnostic evaluation of the entire CT component including the entire colon is therefore an essential part of PET/CT interpretation. Readers of PET-CT need also to be cognizant of the impact of different degrees of rectal and bladder distention can have particularly on pelvic disease coregistration. Similarly caution must be exercised when interpreting liver metastases, as dome lesions can be erroneously projected onto the lung base due to respiratory artifact. False-negative results on FDG PET are particularly common within 4 weeks of chemotherapy. This is thought to be due to chemotherapy, induced cessation of metabolic activity resulting in apoptosis and cell death. A recent study of 224 patients with colorectal hepatic metastases demonstrated that FDG PET within 4 weeks of chemotherapy had a negative predictive value of 13% and a positive predictive value of 94% [63]. 

Fistulous tracts, sinus tracts, and abscesses will typically be associated with intense abnormal FDG tracer uptake, and careful review of the CT images is important in the setting of abnormalities in the region of anastomoses of colon or rectum, and in general in the presacral region in the setting of treated rectal cancer. When a fistula is suspected or questioned, oral contrast becomes particularly important to differentiate neoplasm from physiologic inflammatory tracer uptake.

## 9. Therapy Monitoring with PET-CT

PET-CT is likely the optimal method for detecting recurrence and monitoring treatment response to chemotherapy and radiotherapy in CRC although, in practice, CT is still most commonly used for this purpose [64, 65]. Complete resolution of abnormal glucose metabolism in primary and metastatic lesions of colon cancer can be observed following chemotherapy. FDG-PET can be used to predict response to chemotherapy and radiofrequency ablation in patients with hepatic metastases from CRC [66, 67]. However, many issues including optimal time for imaging, criteria for response, and the decision-making and cost-effective benefits of PET-CT imaging for monitoring therapy response in patients with CRC have not yet been fully established.

## 10. Planning of Radiation Therapy for CRC Using PET-CT

Radiation combined with surgery and chemotherapy is employed for patients with stage II and III rectal cancer. Radiation is associated with a reduced rate of local recurrence but also increases the risks of sexual dysfunction, postoperative mortality in older patients, and perineal dehiscence [68]. Cross-sectional imaging such as CT and MR imaging is the most common method for planning radiation therapy in patients with CRC. These structural imaging techniques provide anatomic delineation of tumor mass and adjacent structures as well as an estimation of tumor volume in three dimensions. However, these techniques have certain challenges including, firstly to differentiate a primary or recurrent malignant lesion from surrounding inflammatory changes or fibrosis, secondly to correctly represent time-averaged position and shape of the tumor due to patient motion, and finally to accurately take into account the metabolic and biologic features of cancer. 

PET-CT imaging for radiation therapy planning has been found to be useful in patients with brain, lung, head, and neck, as well as pelvic cancers (including CRC) [69]. The effect of PET data from an integrated PET-CT system, in addition to the CT images for defining the gross tumor volume (GTV) and as a consequence the clinical tumor volume (CTV) and planning target volume (PTV), has been investigated [69]. GTV increases were observed in three of 6 patients. The mean changes of the GTV were 50%, translating into a 20% change of PTV. PET has the potential to improve the rate of negative margins after surgery, and PET-CT-based radiotherapy planning may help minimize portal field sizes without missing macroscopic tumor tissue adjacent to the primary tumor. PET-defined GTV is also potentially useful for planning the boost volume for adjuvant radiation therapy after tumor resection of rectal cancer if PET-CT is obtained in the radiotherapy position before surgery. PET-CT imaging can be useful for planning laser-induced thermotherapy for liver metastases from CRC [70]. Metastases with increased FDG uptake, which are difficult to localize on PET images alone, can be better identified with fused PET-CT image data. Detection of lesions not seen on CT images can facilitate in planning of laser-induced thermotherapy (and other local treatments) for liver metastases as well as in the identification of patients with extrahepatic metastases, who may not profit from the procedure.

FDG PET and PET-CT can provide added diagnostic information compared with conventional imaging in patients after radiofrequency ablation of liver metastases and can be used to guide repeat ablations [71]. Areas of focal FDG accumulation greater than background activity in the region of the treated lesion are interpreted as being pathologic. On CT an irregular, hypoattenuating area around the margins of the ablated tumor that increases in size on followup scans is interpreted as residual tumor. In one of study 4 of 17 patients whose unresectable liver metastases were treated by RFA, FDG-PET at 1 week and 1 month showed peripheral focal hypermetabolic residue after RFA, whereas CT did not reveal the residual tumor [72]. FDG-PET appears to accurately monitor the local efficacy of RFA for treatment of liver metastases, as it recognizes incomplete tumor ablation earlier than detectable on CT. Langenhoff et al. reported similar findings but we consider comparison of the PET findings with CT essential and should preferably be performed as part of a PET-CT examination [73]. These studies support the use of FDG PET in combination with CT scan in the follow-up of RF ablated colorectal metastases and PET-CT may lead to earlier detection of tumor recurrence than conventional imaging modalities.

## 11. Conclusion

The development of PET-CT has had a considerable impact on the assessment of patients with CRC and it helps decide if treatment intent should be curative or palliative (Table 5). The impact of PET-CT is the greatest for the detection of previously unrecognized sites of metastatic disease, either hepatic or extrahepatic, patients with a rising CEA, and for the detection of local recurrence. A single PET-CT can diagnose local recurrence due to abnormal FDG uptake, whereas serial conventional CTs are often required to characterize recurrence. Similarly, PET-CT does not rely solely on anatomic size to diagnose nodal metastases as is the case with conventional CT. The spatial resolution of PET-CT is approximately 1 cm, and limited FDG uptake by certain tumor cell types such as mucinous adenocarcinoma represents another potential weakness. Increased FDG uptake can occur due to benign disease such as diverticulitis, fistulas, and sarcoid. Future directions for the development of PET-CT will be determined by methods of radiation dose reduction, increasing resolution, newer more specific isotopes, and the continued close working relationship between referring physicians and radiologists.

## Figures and Tables

**Table 1:** Sensitivity and specificity of endoscopic ultrasound, CT, and MRI for perirectal and adjacent organ invasion [10].

	Sensitivity (%)	Specificity (%)
*Perirectal Invasion*		
Endoscopic Ultrasound	90	75
CT	79	78
MRI	82	76
*Adjacent Organ Invasion*		
Endoscopic Ultrasound	70	97
CT	72	96
MRI	74	96

**Table 2:** Sensitivity and specificity of CT, MR, and FDG PET for detecting hepatic metastases from CRC [25].

	Sensitivity (%)	Specificity (%)
CT	84	95
MRI	88	93
FDG PET	94	96
FDG PET/CT	97	97

**Table 3:** Sensitivity and specificity of CT and PET-CT for detection of recurrent or metastatic CRC [29].

	Sensitivity (%)	Specificity (%)
CT	67	63
PET-CT	98	75

**Table 4:** Sensitivity and specificity of FDG PET and PET-CT for detecting pelvic recurrence of CRC [60].

	Sensitivity (%)	Specificity (%)	Accuracy (%)
FDG PET	82	65	93
PET-CT	98	96	74

**Table 5:** Advantages and disadvantages/pitfalls of combined PET-CT.

Advantages	Disadvantages/potential pitfalls
Physiological information with poor spatial resolution of FDG-PET combined with the high spatial resolution of CT yields a study that is better than either component on their own	Potential for false-positive results in the postoperative bed within 4 weeks of surgery
Improved sensitivity and specificity for staging colorectal cancer, detecting local recurrence and metastases	High radiation dose Potential false-negative results with variations in rectal distension and mucinous adenocarcinomas which often do not take up FDG and in lymph nodes measuring less than 1 cm in size
Can be used to predict response to chemoradiotherapy and plan radiotherapy	Potential false-positive results due to the presence of abscesses, sinus, or fistulous tracts
